# High specificity and sensitivity of Zika EDIII-based ELISA diagnosis highlighted by a large human reference panel

**DOI:** 10.1371/journal.pntd.0007747

**Published:** 2019-09-20

**Authors:** Jessica Denis, Sarah Attoumani, Patrick Gravier, Bernard Tenebray, Annabelle Garnier, Sébastien Briolant, Franck de Laval, Véronique Chastres, Gilda Grard, Isabelle Leparc-Goffart, Bruno Coutard, Cyril Badaut

**Affiliations:** 1 Unité de Biothérapies anti-Infectieuses et Immunité, Institut de Recherche Biomédicale des Armées, 1 place du Général Valérie André BP73, Brétigny-sur-Orge Cedex, France; 2 Centre National de Référence des Arbovirus, Institut de Recherche Biomédicale des Armées, Unité d’Arbovirologie HIA Laveran, Marseille, France; 3 Architecture et Fonction des Macromolécules Biologiques, AFMB UMR 7257, Aix Marseille Université/CNRS – Case 932 163, Avenue de Luminy Marseille, France; 4 Unité des Virus Emergents (UVE: Aix-Marseille Univ – IRD 190 – Inserm 1207 – IHU Méditerranée Infection), Marseille, France; 5 Unité de Parasitologie et Entomologie, Département des Maladies Infectieuses, Institut de Recherche Biomédicale des Armées, Marseille, France; 6 Aix Marseille Université, IRD, AP-HM, SSA, UMR vecteurs – Infections Tropicales et Méditerranéennes (VITROME), IHU – Méditerranée Infection, Marseille, France; 7 Service de Santé des Armées, Centre d’Epidémiologie et de Santé Public des Armées, Marseille, France; 8 Aix Marseille Université, INSERM, SESSTIM, Science Economique & Sociales de la Santé & Traitement de l’Information Médicale, Marseille, France; 9 Unité Perception, Département Neuroscience et Sciences Cognitives, Institut de Recherche Biomédicale des Armées, 1 place du Général Valérie André BP73, Brétigny-sur-Orge, France; University of California San Francisco, UNITED STATES

## Abstract

**Background:**

Zika virus (ZIKV) and Dengue virus (DENV) are often co-endemic. The high protein-sequence homology of *flaviviruses* renders IgG induced by and directed against them highly cross-reactive against their antigen(s), as observed on a large set of sera, leading to poorly reliable sero-diagnosis.

**Methods:**

We selected Domain III of the ZIKV Envelope (ZEDIII) sequence, which is virus specific. This recombinant domain was expressed and purified for the specific detection of ZEDIII-induced IgG by ELISA from ZIKV-RT-PCR-positive, ZIKV-IgM-positive, *flavivirus*-positive but ZIKV-negative, or *flavivirus*-negative sera. We also assessed the reactivity of ZEDIII-specific human antibodies against EDIII of DENV serotype 4 (D4EDIII) as a specific control. Sera from ZEDIII-immunized mice were also tested.

**Results:**

Cross-reactivity of IgG from 5,600 sera against total inactivated DENV or ZIKV was high (71.0% [69.1; 72.2]), whereas the specificity and sensitivity calculated using a representative cohort (242 sera) reached 90% [84.0; 95.8] and 92% [84.5; 99.5], respectively, using a ZEDIII-based ELISA. Moreover, purified human IgG against D2EDIII or D4EDIII did not bind to ZEDIII and we observed no D4EDIII reactivity with ZIKV-induced mouse polyclonal IgGs.

**Conclusions:**

We developed a ZEDIII-based ELISA that can discriminate between past or current DENV and ZIKV infections, allowing the detection of a serological scar from other *flaviviruses*. This could be used to confirm exposure of pregnant women or to follow the spread of an endemic disease.

## Author summary

The serological detection of Zika virus (ZIKV) is a challenge, as ZIKV infection generally leads to an immune response with a high level of cross reactivity against related viruses, such as Dengue virus. Although seroneutralization assays are the gold-standard to address specificity, a rapid and cost-effective detection assay with good specificity and sensitivity could be used for first-line screening. We used a large cohort to define a set of human reference sera to validate an ELISA based on a recombinant ZIKV antigen. The assay showed 90% specificity and 92% sensitivity, providing a good basis for the development of diagnostic assays. Characterization of both DENV-EDIII-purified human and murine IgG induced by ZIKV infection or ZEDIII, respectively, confirmed the good specificity of the antigen.

## Introduction

Zika virus (ZIKV) was isolated in 1947 in the Zika forest in Uganda [1] and has been responsible for sporadic cases for several decades. Epidemics in Yap State, Micronesia (2007) [2], French Polynesia (2013) [3], and more recently the Americas 2015 [4], have dramatically changed its status. The association of ZIKV infection with Guillain-Barré syndrome [5] and severe outcomes during pregnancy, including microcephaly in fetuses and neonates [6, 7], have been brought to light during the last outbreaks. ZIKV can be sexually transmitted. This is uncommon for *flaviviruses*, which are arthropod-borne viruses (*arbovirus*) [8]. Infective ZIKV particles have also been found in breast milk but whether neonatal infection or perinatal transmission can occur is unclear [9]. Due to the ZIKV threat, the World Health Organization (WHO) declared a Public Health Emergency of International Concern on the 1^st^ February 2016 [10].

According to WHO recommendations, the diagnosis is based on detection of the ZIKV genome by real-time reverse transcription PCR, serology, and neutralization assays, such as plaque-reduction neutralization tests (PRNT) [11]. However, induced antibodies can show high cross-reactivity between antigens of the same family, as *Flaviviruses* are phylogenetically very close. Moreover, Dengue virus (DENV) and ZIKV can co-circulate [12]. In addition, cross-seroneutralization of DENV and ZIKV, described recently [13], further complicates the development of relevant target antigens for reliable serological diagnosis. Several immunodiagnostics based on IgM detection have been developed [14, 15]. However, most cases are asymptomatic [16] and ZIKV-IgG detection is relevant for dating or confirming infections, especially those of pregnant women, retrospective diagnosis for evaluating transmission intensity to decide on the use of a new prophylaxis, or confirming future ZIKV protection by vaccination.

Domain III of the ZIKV-envelope protein (EDIII) shares 29% amino-acid identity with DENV-EDIII and 90% of EDIII-antibodies (EDIII-Abs) elicited by ZIKV infection are virus specific [17]. ZIKV EDIII-Abs tested in ELISA do not bind to DENV-2 or West-Nile virus (WNV) -EDIII [18]. Studies in mice have shown that treatment with EDIII-Abs protect against ZIKV infection [19] by neutralizing the virus [18].

In this context, we produced a recombinant ZIKV-EDIII protein (ZEDIII) and assessed its recognition by IgG. We constructed four representative panels of reference sera, (1) ZIKV-RT-PCR-positive, (2) ZIKV-IgM-positive, (3) *flavivirus*-positive but ZIKV-negative, and (4) *flavivirus*-negative, from more than 5,000 sera serodiagnosed at the French National Reference Center for Arboviruses (NRC). The objective was to evaluate a ZEDIII-based ELISA for its specificity (ability to detect IgG raised after infection by ZIKV, but not other flaviviruses) and sensitivity (ability to detect ZIKV-specific IgG in the serum of a patient confirmed to be ZIKV-positive). We thus designed three experiments to evaluate: (1) the specificity and sensitivity of recognition by the IgG of cohort sera, (2) potential purified DENV-IgG cross-reactivity, and (3) the specificity of ZEDIII-induced IgG. The specificity of the assay represents its ability to not detect false-positive sera, that is positivity due to cross-reactive antibodies that are able to recognize a common antigen with the same affinity shared by various viruses.

## Materials and methods

### Ethics statement

As French National Reference center, all samples used in this study were send to the laboratory for arboviruses diagnosis including the specificity of the antibodies response against different flaviviruses, as Zika virus. For this study, samples were analyzed anonymously. All samples sent in the laboratory are associated with a file with clinical data, travel, date of symptoms and also the consent to used the end of tube for technic development or comparison of diagnosis technics.

### Human ethics

All sera were submitted to the French National Reference Center for arboviruses (NRC, Marseille) for routine diagnosis and stored at −20°C in an anonymized biobank before testing. No specific sampling dedicated to the study was performed. There were no legal or ethical restrictions for sample use. Associated documents contained the following information: date of birth, gender, date, nature of sampling, place of stay and return date, symptom onset date, clinical symptoms, and clinical diagnosis results.

Ethical approval of the ZIFAG cohort was given by the “Comité de Protection des Personnes Sud-Mediterranée” corresponding to the “Etude descriptive prospective de la maladie a virus Zika au sein de la communaute de defense des Forces Armees en Guyane—ZIFAG” and was registered on 29 February 2016 as RCB: 2016-A00394-47. Written consent was obtained from all participants [20].

### Clinical samples

A total of 5,600 sera from the NRC Arbovirus serum bank were serodiagnosed by ELISA using inactivated virus as antigen. This group was selected to follow antibody cross-reactivity between ZIKV and DENV. Eighty-one sera were not tested for the ZIKV-IgM response.

A set of sera was selected (n = 242) from the 5,600 of the NRC Arbovirus serum bank, as well as a set from the ZIFAG cohort, and divided into four groups based on their genome detection, reaction against inactivated arboviruses, and epidemiological data (Table 1, SD1 Fig). The “ZIKV-RT-PCR-positive” group, from the ZIFAG one-year clinical follow-up cohort, is composed of 43 sera collected in French Guyana during the 2016 ZIKV outbreak [20, 21]. The ZIKV genome was detected by RT-PCR during the acute phase and tested sera were obtained during convalescence: 6 to 195 days post symptoms onset (DPSO). Eighty-four percent of this group (n = 36) was sampled during the first month post symptoms onset: two sera were obtained less than 10 days DPSO and 34 between 13 and 25 DPSO. The other sera (n = 7) were obtained between 35 and 195 DPSO [21]. The “ZIKV-IgM-positive” group was composed of 50 randomly selected sera, collected mainly from the Caribbean Islands or another DENV-endemic area during the 2015–2016 ZIKV epidemic (total n = 241), based on the following criteria: ZIKV-IgM-positive, DENV-IgM negative, and ZIKV-IgG positive, all tested sera neutralizing ZIKV in PRNT assays. “*Flavivirus”* sera (n = 99) group was selected to be *flavivirus-*positive but not ZIKV-positive (total n = 797). All were *flavivirus-*IgG positive and sampled between 2013 and 2014 in Guadeloupe, Martinique, or Saint Martin, DENV-endemic areas, where and when ZIKV was not circulating, assuming that these patients were not ZIKV infected. The “Negative” sera group was composed of 50 negative sera that were both IgM- and IgG-negative for DENV, WNV, Chikungunya virus, Encephalitis St Louis virus, ZIKV, Toscana virus, and Rift Valley fever virus. The ZIKV-IgM-positive, *flavivirus* and negative groups were representative of the entire parent groups in terms of their mean age and immune responses. We determined the sensitivity of the ZEDIII-based ELISA with the ZIKV-RT-PCR-positive group and compared it to that of the ZIKV-IgM-positive group, and the specificity to that of the *flavivirus*-positive group. The serology of the samples was tested upon their arrival to the arbovirus NRC and then stored at -20°C. The antibodies were stable over time and no difference in response was observed several months or years after freezing.

**Table 1:** Description of the sera for NRC, ZIKV, *Flavivirus*, and Negative groups.

Groups	NRC	ZIKV-RT-PCR-positive	ZIKV-IgM-positive	*Flavivirus*	Negative
Number, n	5600	43	50	99	50
Gender, n women (%), n men (%), n ND (%)	3624 (65%), 1867 (33%), 109 (2%)	12 (28%), 31 (72%)	42 (84%), 8 (16%)	61 (62%), 37 (37%), 1 (1%)	22 (44%), 28 (56%)
Age in years, median, IQR, (range), n ND (%)	35, 29–49, (0–91)	39, 32–43, (21–65)	36, 30–53, (9–80)	51, 36–65, (6–84)	46, 28–58, (2–78), 1 (2%)
DPSO, median, IQR, (range), n ND (%)	-	17, 15–22, (6–195)	26, 17–55, (6–139), 3 (6%)	20, 11–48, (8–99), 32 (32%)	17, 12–27, (7–175), 11 (22%)
ZIKV seroneutralizer, n (%), n ND (%)	-	-	35 (70%), 15 (30%)	-	-
Sera origins					
America, n (%)	3523 (62.9%)	43 (100%)	48 (96%)	-	22 (44%)
Pacific region, n (%)	353 (6.3%)	-	1 (2%)	-	2 (4%)
Asia, n (%)	494 (8.8%)	-	0 (0%)	-	9 (18%)
Africa, n (%)	407 (7.3%)	-	0 (0%)	-	13 (26%)
Europe, n (%)	745 (13.3%)	-	1 (2%)	-	4 (8%)
ND, n (%)	78 (1.4%)	-	0 (0%)	-	0 (0%)
Guadeloupe, n (%)	-	-	-	51 (52%)	-
Martinique, n (%)	-	-	-	38 (38%)	-
Saint-Martin, n (%)	-	-	-	10 (10%)	
DENV/ZIKV Spearman r (p < 0.0001) for IgG	0.7986	-	0.8417	0.7811	NS

Abbreviations: NRC, French National Reference Center for arboviruses; ND, not documented; IQR, inter-quartile range; DPSO, day-post symptom onset; DENV, Dengue virus; NS, not significant.

### EDIII production and purification

Sequences of the Asian ZIKV strain from the French Polynesia outbreak of 2013, DENV2, the most frequently detected serotype by the NRC for Arbovirus, and DENV4, the DENV strain phylogenetically closest to ZIKV (accession numbers AHZ13508, P09866, and M29095, respectively), were selected for EDIII production using the structure of the ZIKV envelope protein (PDB 5JHM). The ZEDIII, D2EDIII, and D4EDIII coding sequences, optimized for production in *E*.*coli*, were synthesized in fusion with a His-tag coding sequence at their 5’ end by GenScript. The sequences were then cloned into pET-24a or pET-19b plasmids (Novagen). The recombinant proteins were produced in *E*. *coli* T7 Iq Express (New England Biolabs) and purified under denaturing conditions prior to *in vitro* refolding according to a protocol described previously [22]. The purity, conformation, and homogeneity of folding of the protein were controlled by size-exclusion chromatography coupled to multi-angle light-scattering analysis (SEC-MALS) and Coomassie blue-stained SDS PAGE gels.

### IgG purification from EDIII-positive sera using recombinant ZEDIII, D2EDIII, or D4EDIII

IgG were purified from pools of 20 sera from the ZIKV-positive group against ZEDIII, and 20 positive sera from the *flavivirus*-positive but ZIKV-negative groups against D2EDIII or D4EDIII (25 μL per sera for a total of 500 μL per pool). Antibody purification was carried out in two steps, first on a protein-G column and then on a column with immobilized recombinant EDIII. For the immobilization of EDIII, 1 mg of recombinant ZEDIII, D2EDIII, or D4EDIII in 2.5 mL phosphate-buffered saline (PBS) was covalently bound to an NHS-activated column following the manufacturer’s protocol (GE Healthcare). Four milligrams of protein-G-purified antibodies from EDIII positive sera in 4 mL were then loaded onto the EDIII-coupled columns. After washes with PBS, EDIII-specific antibodies were eluted from the column with 1 mL 0.1 M glycine (pH 2.7) and neutralized with 100 μL 1 M Tris (pH 9).

### Virus production, inactivation, and purification

Vero cells were inoculated with 0.01 MOI ZIKV (African ZIKV strain, accession number ArB41644) or DENV2 (Martinique DENV2 98–703 strain of 1998, accession number AF208496) and grown in Dulbecco’s modified Eagle medium (DMEM) complemented with 2% heat-inactivated fetal calf serum (FCS) at 37°C in 5% CO2 for 3.5 (ZIKV) or 7 (DENV2) days. The time of growth depended on the virus. Culture supernatants were centrifuged, and viral particles precipitated with polyethylene glycol 6000 (PEG 6000) and NaCl. The precipitates were washed and resuspended in PBS/Hepes solution. This viral solution was inactivated with beta-propiolactone.

### Mouse immunization

Six-to-eight-week old female AG129 mice, weighing approximately 20 g at the start of the study, were subcutaneously injected with 10 μg ZEDIII in 20 μL PBS on day 0 and received a boost on days 15 and 30. Blood was collected on day 46 via the retro-orbital route.

All procedures were in accordance with the guidelines set by the Noble Life Sciences (NLS) Animal Care and Use Committee. Noble is fully accredited by the Association for the Assessment and Accreditation of Laboratory Animal Care International (AAALAC). Veterinary care for all lab animals was in accordance with the Public Health Service Policy, U.S. Dept. of Agriculture (USDA) and AAALAC International requirements. All mice were bred at B&K Universal Laboratories under specific pathogen-free conditions. All experiments were carried out at Integrated BioTherapeutics, Inc.

### ELISA responses of sera and purified IgG to EDIII

96-well plates (Maxisorp, NUNC-Immuno Plate) were coated with 200 ng/well of recombinant protein diluted in PBS and incubated over night at 4°C. After removing antigen, wells were blocked with blocking buffer (3% milk in PBS) for 1 h at 25°C. After two washing steps with washing buffer (1% Tween 20 in PBS), they were incubated with 100 μL diluted human serum samples (1:500), diluted purified human IgG (0.15 or 0.06 μg/mL), or sera from vaccinated mice diluted (1:25) in dilution buffer (3% milk and 0.1% Tween 20 in PBS), then incubated 1 h at 25°C. After four washing steps, the plates were incubated with horseradish peroxidase-conjugated anti–human IgG (1:10,000) (Jackson Laboratories, Immuno Research) or horseradish peroxidase-conjugated anti–mouse IgG (1:40,000) (Sigma-Aldrich) diluted in dilution buffer for 1 h at 25°C. After four washing steps, TMB (KPL, TMB microwell Peroxidase Substrate System) was added and the reaction stopped after 5 min with 50 μL 0.25M H_2_SO_4_ per well. The optical density (OD) was read at 450 nm and the final values, OD ratio (ODr), obtained by dividing the average OD of duplicate wells from that of the corresponding blank non-coated wells. The threshold of positivity was calculated following the equation: m + 3σ (m = mean, σ = standard deviation, α (error) = 1%) on the ODr of the negative-sera group. Self-recognition of human purified IgG ODr was normalized to 100% for each EDIII.

### ELISA responses of sera to inactivated viruses

96-well plates (Maxisorp, NUNC-Immuno Plate) were coated with inactivated viruses diluted (1:200) in PBS and incubated over night at 4°C. After removing antigen, wells were blocked with blocking buffer (3% milk and 1% sodium azide in PBS) for 1 h at room temperature (RT) in the dark. After two washing steps with washing buffer (0.05% Tween 20 in PBS), they were incubated with 100 μL human serum samples diluted (1:500) in dilution buffer (3% milk, 0.05% Tween 20, and 1% sodium azide in PBS), then incubated 1 h at 41°C. After four washing steps, the wells were incubated with human secondary antibody, horseradish peroxidase-conjugated anti–human IgG (1:10 000) (Jackson Laboratories, Immuno Research), diluted in dilution buffer and incubated 1 h at 41°C. After four washing steps, TMB (KPL, TMB microwell Peroxidase Substrate System) was added and the reaction stopped after 5 min with 50 μL 0.25M H2SO4 per well. The optical density (OD) was read at 450 nm and final values were obtained by dividing the average OD of duplicate wells from that of the corresponding blank wells coated with negative antigen. The threshold of positivity was fixed to an ODr of 3.

### ELISA responses of sera to NS1 antigen (Euroimmun kit)

IgG responses of human sera samples were tested against NS1 (Euroimmun kit) following the manufacturer’s protocol (Euroimmun). Ambiguous results were considered to be negative.

### Statistical analysis

Statistical analyses were performed with Prism 6 (GraphPad). IgM and IgG responses to DENV and ZIKV were correlated and R-values determined by the Spearman rank correlation test with n = 5,600.

The IgG response data of the selected groups (ZIKV-RT-PCR-positive, n = 43; ZIKV-IgM-positive, n = 50; *Flavivirus*, n = 99; Negative, n = 50) were plotted showing the median, 25^th^, and 75^th^ percentiles. The non-parametric Kruskal-Wallis test with Dunn’s correction was used to identify the differences between the medians of the IgG responses with two-tailed and alpha = 0.01 for each test. The two-tailed Z-test with alpha = 0.05 was used to compare the difference in sensitivity of the ZEDIII- and NS1-based ELISAs according to the group or cohort used. The overlap of the IC 95% was assessed to compare the difference of misdiagnoses between the ZEDIII- and NS1-based ELISAs. The non-parametric Wilcoxon rank-sum test was used to compare the difference of the mouse IgG responses to ZEDIII or D4EDIII.

The study was performed in compliance with the STAndards for Reporting of Diagnostic accuracy (STARD) statement [23].

## Results

### Cross-reactivity of IgM and IgG between Dengue, West-Nile, and Zika Viruses

**Fig 1:**
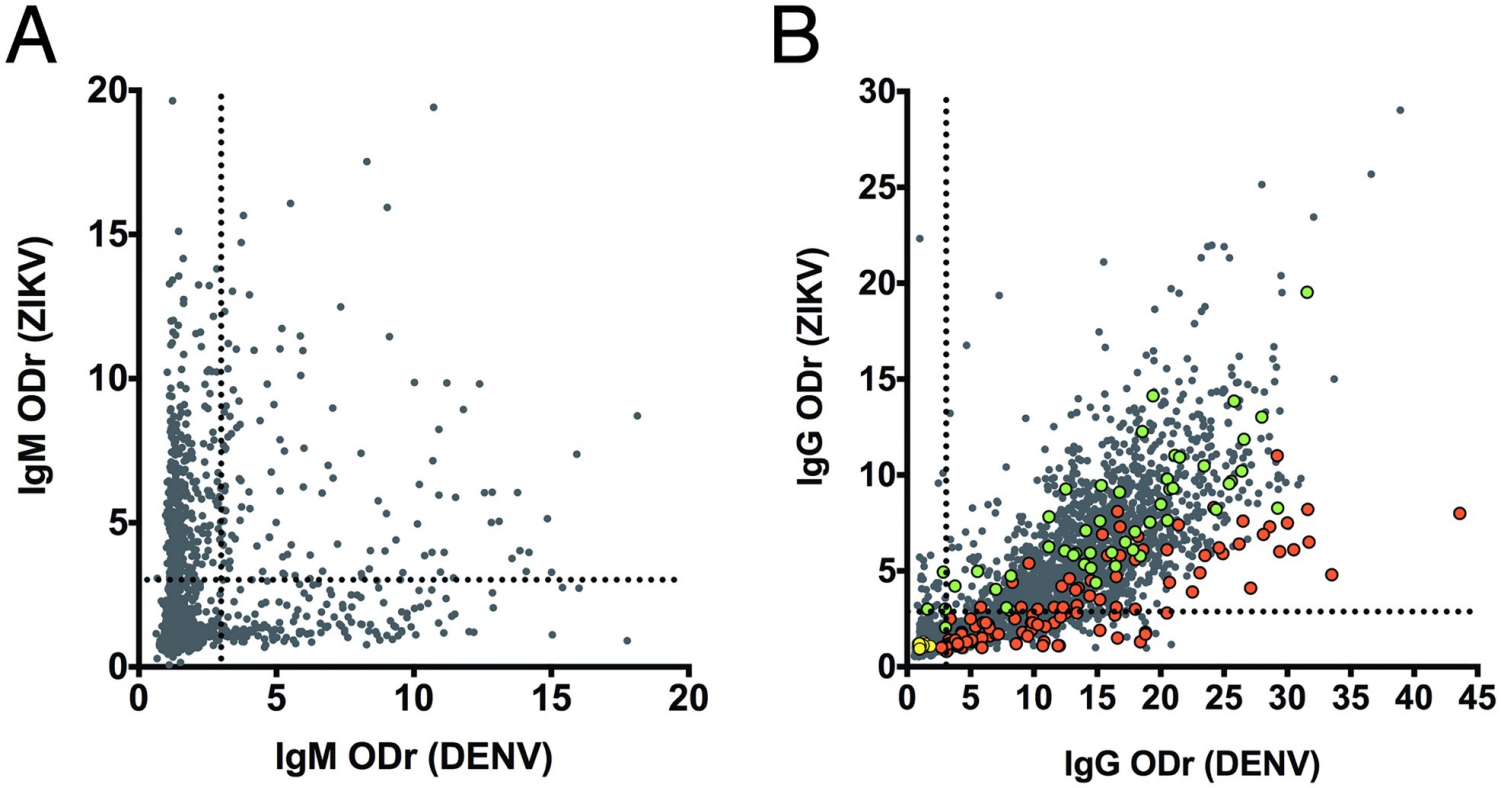
Reactivity of sera against inactivated Dengue and Zika viruses by ELISA. IgM (A) and IgG (B) Optical Density ratio (ODr = OD (target) / OD (noise)) against DENV (abscissa) and ZIKV (ordinate) obtained by ELISA. Values are positive if ODr is ≥ 3 (dotted line) in an ELISA assay using total inactivated virus as target. The IgM group (n = 5,519) is included in the IgG group (n = 5,600). Three groups were assembled: ZIKV-IgM-positive (green, n = 50), *flavivirus* (red, n = 99), and negative (yellow, n = 50) groups.

IgM cross-reactivity [95% Confidence Interval] between related *flaviviruses* (Fig 1A, Table 2) represented 23.8% [20.4; 27.2] of the sera (n = 121) that reacted against at least one virus (n = 606). In contrast, IgG cross-reactivity [95% Confidence Interval] (Fig 1B) represented 71.0% [69.1; 72.2] of the sera (n = 1,485) that reacted against at least one virus (n = 2,091). This cross-reactivity was evaluated by calculating the Spearman r correlation (p < 0.0001; r(IgM) = 0.4191, r(IgG) = 0.7986). The r value for IgG indicates a strong correlation between the recognition of ZIKV and DENV (Table 1). We obtained similar results concerning the cross-reactivity of IgM (11.6% [8.3; 14.9]; n = 42) and IgG (79.7% [77.4; 82]; n = 971) against WNV and ZIKV (Table 3).

**Table 2:** Percentage of sera that recognize, or not, DENV and ZIKV.

	IgM (n = 5,519)		IgG (n = 5,600)	
n, % (%)	DENV-	DENV+	DENV-	DENV+
ZIKV+	**341**, 6.2% (56.3%)	**121**, 2.6% (23.8%)	**25**, 0.4% (1.2%)	**1,485**, 26.5% (71.0%)
ZIKV-	**4,913**, 89.0%	**144**, 2.2% (20%)	**3,509**, 62.7%	**581**, 10.4% (27.8%)

Abbreviations: ZIKV, Zika virus; DENV, Dengue virus.

n is the number of sera recognizing (+) or not (-) DENV and/or ZIKV. The percentage of sera (IgM or IgG) recognizing at least one of the viruses is shown in the brackets.

**Table 3:** Percentage of sera that recognize, or not, WNV and ZIKV.

	IgM (n = 3,360)		IgG (n = 3,441)	
n, % (%)	WNV-	WNV+	WNV-	WNV+
ZIKV+	**241**, 7.2% (67.0%)	**42**, 1.2% (11.6%)	**19**, 0.5% (1.5%)	**971**, 28.2% (79.7%)
ZIKV-	**3,000**, 89.3%	**77**, 2.3% (21.4%)	**2,222**, 64.6%	**229**, 6.7% (18.8%)

Abbreviations: WNV, West-Nile Virus; ZIKV, Zika virus.

n is the number of sera recognizing (+) or not (-) WNV and/or ZIKV. The percentage of sera (IgM or IgG) recognizing at least one of the viruses is shown in the brackets.

IgG from the sera of the ZIKV-positive group and the *flavivirus*-positive but ZIKV-negative group (colored dots, Fig 1B) cross reacted with DENV and ZIKV similarly to all 5,600 sera: 68.7% *versus* 71% of IgG, showing that these two panels are representative of the 5,600 sera (Table 1).

### Domain III sequence selection and variability

**Fig 2:**
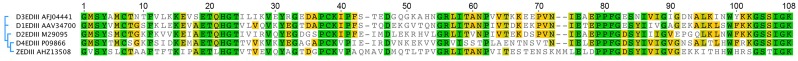
Similarity of domain III of the envelope protein (EDIII). EDIII sequences are aligned. Green: 100% similar; khaki: 80 to 100% similar; orange: 60 to 80% similar; grey: less than 60% similar. Numbering is relative to that of ZEDIII.

**Fig 3:**
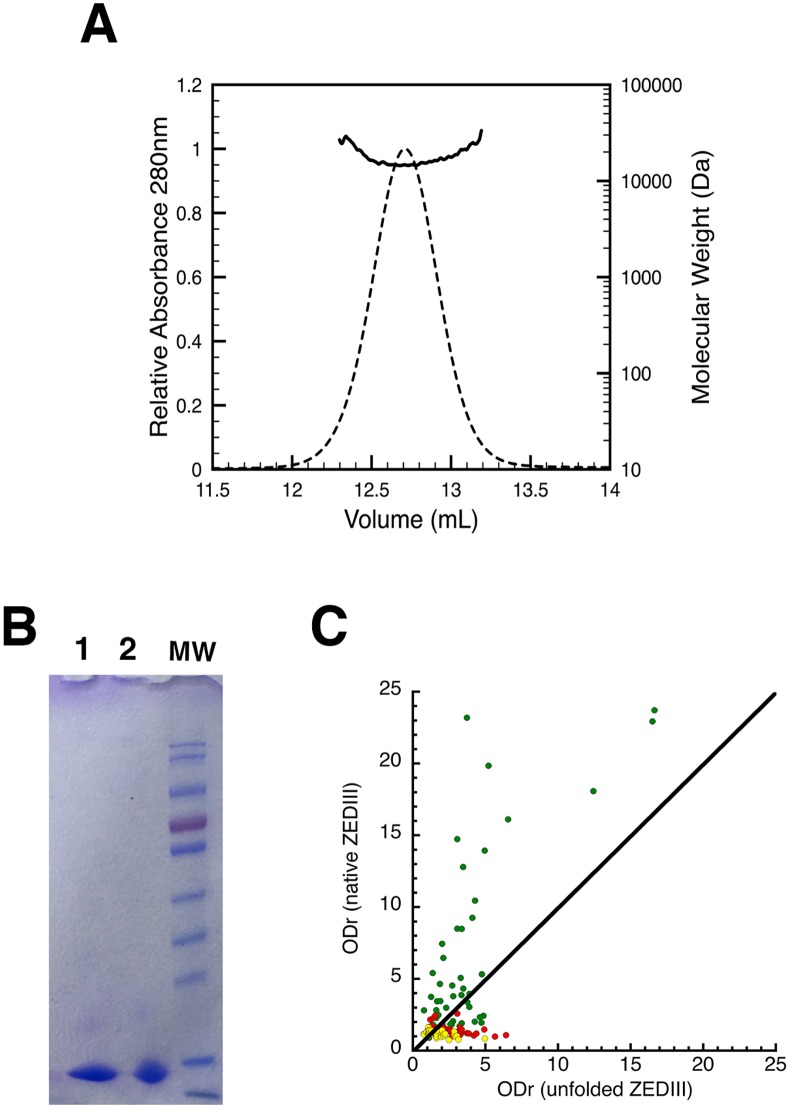
Characterization of refolded ZEDIII. (A) Raw data of the analytical size-exclusion chromatography of ZEDIII followed by MALS/UV/refractometry. The abscissa and ordinate axes correspond to the elution volume and the molecular weight (Da) obtained by MALS, respectively. The OD_280nm_ is represented by the non-continuous black trace and the molecular weight by the continuous black trace. The value of the measured molar mass (14.5 kDa) at the volume corresponding to the top of the peak is reported. The population of ZEDIII with a molar mass of 14.5 kDa is more than 95%. (B) Purity of ZEDIII after refolding and purification analyzed on an SDS-PAGE gel stained with Coomassie blue. MW: molecular weight (in kDa). Lane 1: ZEDIII under reducing conditions (DTT); Lane 2: ZEDIII under non-reducing conditions. (C) Recognition of linear and conformational epitopes by ZIKV-IgM-positive (green, n = 50), *flavivirus* (red, n = 50), and negative (yellow, n = 50) groups by ELISA.

The ZEDIII amino-acid sequence shares 46.3% and 47.2% identity and 64.8% homology with the amino-acid sequences of D2EDIII and D4EDIII, respectively (Fig 2). The DENV2 and DENV4 EDIII amino-acid sequences share 61% identity and 78.1% homology. The purified EDIIIs analyzed by SEC-MALS and Coomassie blue-stained SDS Page gels showed the elution of a pure protein in a single and symmetric peak with an average molecular weight of 14.5 kDa and a polydispersity of 1.01 (Fig 3A and 3B). The presence and importance of the conformational epitopes for ZEDIII recognition was highlighted by the loss of recognition of ZIKV-positive sera against chemically and thermally denatured ZEDIII and a gain of recognition of flavivirus-positive but non ZIKV-positive or negative sera (Fig 3C).

### ELISA responses of IgG against viral or recombinant protein targets

**Fig 4:**
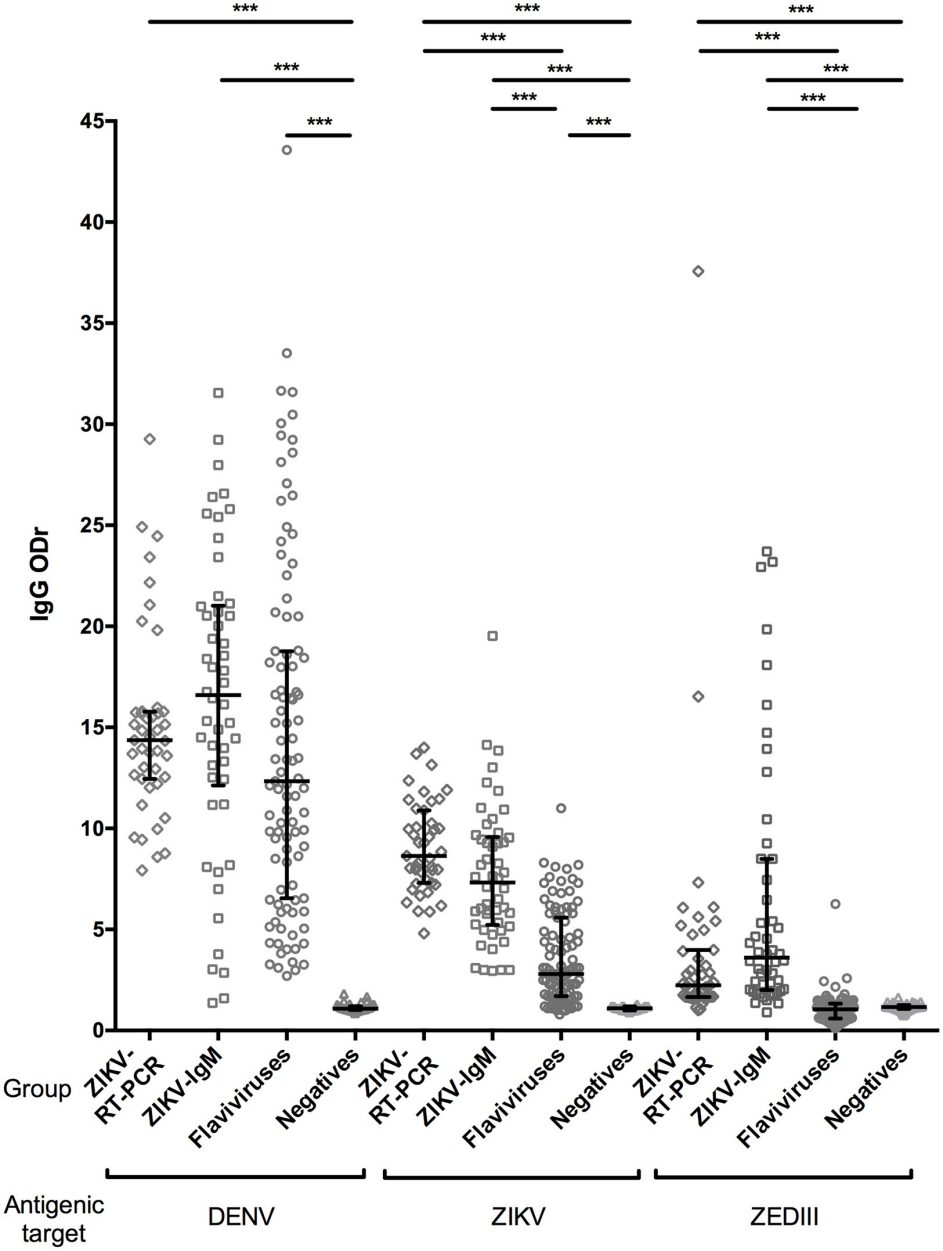
Detection of inactivated ZIKV or DENV or ZDIIIE antigens by the four reference panels. An ELISA experiment with 242 sera selected for their status (ZIKV-RT-PCR-positive (n = 43), ZIKV-IgM-positive (n = 50), *flavivirus* (n = 99), or negative (n = 50)) was performed against ZIKV, DENV, or ZEDIII antigens in duplicate. The top, bottom, and middle lines correspond to the 75^th^, 25^th^, and 50^th^ (median) percentiles, respectively. ***K, p < 0.0001.

We investigated the reactivity of IgG of the four sera groups against inactivated DENV, ZIKV, ZEDIII, and NS1 proteins (Fig 4 and [21]) by ELISA. The ZIKV-RT-PCR-positive, ZIKV-IgM-positive, and *flavivirus* groups recognized DENV with medians of 14.4, 16.6, and 12.3 ODr, respectively, with no statistical difference (K = 5.02, p = 0.0811), whereas no negative serum samples recognized DENV (median ODr of 1.1). The ZIKV-RT-PCR-positive, ZIKV-IgM-positive, and *flavivirus* groups recognized ZIKV (median ODr of 8.6, 7.3, and 2.8, respectively). The levels of recognition between the ZIKV-RT-PCR-positive or ZIKV-IgM-positive groups and flavivirus group were statistically different (Mean rank diff. = 89.61, p < 0.0001; Mean rank diff. = 64.98, p < 0.0001) but similar for the ZIKV-RT-PCR-positive and ZIKV-IgM-positive groups (Mean rank diff. = 24.63, p = 0.0993). The negative group did not recognize ZIKV (median ODr of 1.1). The difference between the ability of the ZIKV-RT-PCR-positive and *flavivirus* groups (median ODr of 2.2 and 1.1, respectively) or ZIK-IgM-positive and *flavivirus* groups (median ODr of 3.6 and 1.1, respectively) to recognize ZEDIII was highly significant (Mean rank diff. = 103.3, p < 0.0001, Mean rank diff. = 123.2, p < 0.0001, respectively), whereas there was no statistical difference between the *flavivirus* and negative sera (median ODr of 1.1 and 1.2; respectively, Mean rank diff. = -13.75, p > 0.9999) or the ZIKV-RT-PCR-positive and ZIKV-IgM-positive sera (median ODr of 2.2 [1.7; 4.0] and 3.6 [2.0; 8.5]; respectively, Mean rank diff. = -19.82, p > 0.9999). The median of the OD obtained for the ZIKV-RT-PCR-positive and ZIKV-IgM-positive sera against ZEDIII was 0.12 (IQR [0.09; 0.24] min = 0.06; max = 1.96) and 0.29 (IQR [0.17; 0.64] min = 0.07; max = 2.10) respectively, whereas the median OD obtained against the blank was 0.05 (IQR [0.05; 0.06] min = 0.05; max = 0.08) and 0.08 ([0.07; 0.10], min = 0.05, max = 0.23), respectively.

With a calculated positive threshold based on the negative-group values (m + 3σ) of 1.54 (m = 1.17 and σ = 0.13), and tolerating a 1% error rate, one serum (2%) of the negative sera was positive against ZEDIII and 49 (98%) were negative. The smallest OD value considered to be positive with this positive threshold was 0.085, with a blank of 0.055, and the highest OD value was 2.12. The sensitivity was calculated as the number of positive sera from the ZIKV-RT-PCR-positive or ZIKV-IgM-positive groups, of which 39 of 43 (90.7%) or 46 of 50 (92.0%), respectively, were positive against the ZEDIII-based assay (Table 4) and four (9.3% or 8.0%, respectively) did not react. The sensitivity [95% confidence interval] was thus 90.7% [82.0; 99.4] or 92.0% [84.5; 99.5], respectively. There was no difference in sensitivity between these two groups (Z-test, p = 0.2219). The specificity was calculated as the negative sera in the *flavivirus* group (n = 99) or in both the *flavivirus* group plus the negative group (n = 149). Ten sera (10.1%) of the *flavivirus* group or 11 sera (7.4%) of the *flavivirus* group plus the negative group were positive against ZEDIII and 89 or 138 were negative (89.9% and 92.6%, respectively). Thus, the specificity [95% confidence interval] was 89.9% [84.0; 95.8] or 92.6% [84.4; 100]. These groups were also tested against NS1 protein using the Euroimmun kit. We determined a specificity of 83.8% [76.8; 91.2] with the flavivirus group and up to 89.3% [84.3; 94.3] with the *flavivirus* group plus the negative group and a sensitivity of 88.4% [78.8; 98.0] with the ZIKV-RT-PCR-positive group and 96.0% [90.6; 100.0] with the ZIKV-IgM-positive group (Table 4). There was no statistical difference in sensitivity between these two groups (Z-test, p = 1.3535). The specificity obtained with the NRC ELISA against ZIKV was 46.5% [36.7; 56.3]; the sensitivity was 100% with the ZIKV-RT-PCR-positive group and 96.0% [90.6; 100] with ZIKV-IgM-positive group.

**Table 4:** Number of positive results obtained by ELISA against DENV, ZIKV, ZEDIII, and NS1 by the ZIKV-RT-PCR-positive, ZIKV-IgM-positive, *Flavivirus*, and negative groups.

Antigenic target	DENV	ZIKV	ZEDIII	ZIKV NS1 (Euroimmun kit)
ZIKV-RT-PCR-positive sera (n = 43), number of positive responses, n (%) [95% confidence interval]	43 (100%)	43 (100%)	39 (90.7%) [82.0; 99.4]	38 (88.4%) [78.8; 98.0]
ZIKV-IgM positive sera (n = 50), number of positive responses, n (%) [95% confidence interval]	48 (96%) [90.6; 100]	48 (96%) [90.6; 100]	46 (92%) [84.5; 99.5]	48 (96%) [90.6; 100]
*Flavivirus* sera (n = 99), number of positive responses, n (%) [95% confidence interval]	99 (100%)	53 (53%) [43.2; 62.8]	10 (10%) [4.1; 15.9]	16 (16%) [8.8; 23.2]
Negative sera (n = 50), number of positive responses, n (%) [95% confidence interval]	0 (0%)	0 (0%)	1 (2%) [0; 5.9]	0 (0%)

Abbreviations: DENV, Dengue virus; ZIKV, Zika virus; ZEDIII, ZIKV envelope protein domain III, NS1, non-structural protein 1.

We compared the reliability of the ZEDIII- and NS1-based ELISA by calculating the positive predictive value (PPV), the false discovery rate (FDR), the negative predictive value (NPV), and the false omission rate (FOR) within a ‘hypothetical population’ with a given prevalence of 33.5% (50/149), composed of ZIKV-IgM-positive (sensitivity identical to that of the ZIKV-RT-PCR-positive group) and *flavivirus* groups (n = 149; Table 5). The likelihood of being positive when detected as positive (PPV) was 82.1% [72.1; 92.1] with the ZEDIII- and 75.0% [64.4; 85.6] with the NS1-based ELISA, whereas the likelihood of being negative when detected as negative (NPV) was 95.7% [91.6; 99.8] and 97.6% [94.3; 100] with the ZEDIII- and NS1-based assays, respectively. The likelihood of being positive when not detected (FOR) was 4.3% [0.2; 8.4] and 2.4% [0; 5.7] with the ZEDIII- and NS1-based ELISAs, respectively, and the likelihood of being negative when detected as positive (FDR) was 17.9% [7.9; 27.9] and 25.0% [14.4; 35.6] with the ZEDIII- and NS1-based ELISAs, respectively.

**Table 5:** Comparison of the positive and negative predictive values between the ZEDIII- and NS1-based-ELISAs in a hypothetical population with 33.5% prevalence.

Antigenic target	ZEDIII	NS1
**PPV, %, [95% CI]**	46/56, 82.1, [72.1; 92.1]	48/64, 75.0, [64.4; 85.6]
**FOR, %, [95% CI]**	4/93, 4.3, [0.2; 8.4]	2/85, 2.4, [0; 5.7]
**NPV, %, [95% CI]**	89/93, 95.7, [91.6; 99.8]	83/85, 97.6, [94.3; 100]
**FDR, %, [95% CI]**	10/56, 17.9, [7.9; 27.9]	16/64, 25.0, [14.4; 35.6]
**P misdiagnosis, %, [95% CI]**	14/149, 9.4, [4.7; 14.1]	18/149, 12.1, [6.9; 17.3]

Abbreviations: ZEDIII, ZIKV envelope protein domain III; NS1, non-structural protein 1; PPV, positive predictive value; FOR, false omission rate; NPV, negative predictive value; FDR, false discovery rate; CI, confidence interval

### Evaluation of the avidity and cross-reactivity of ZEDIII binding IgG

**Fig 5:**
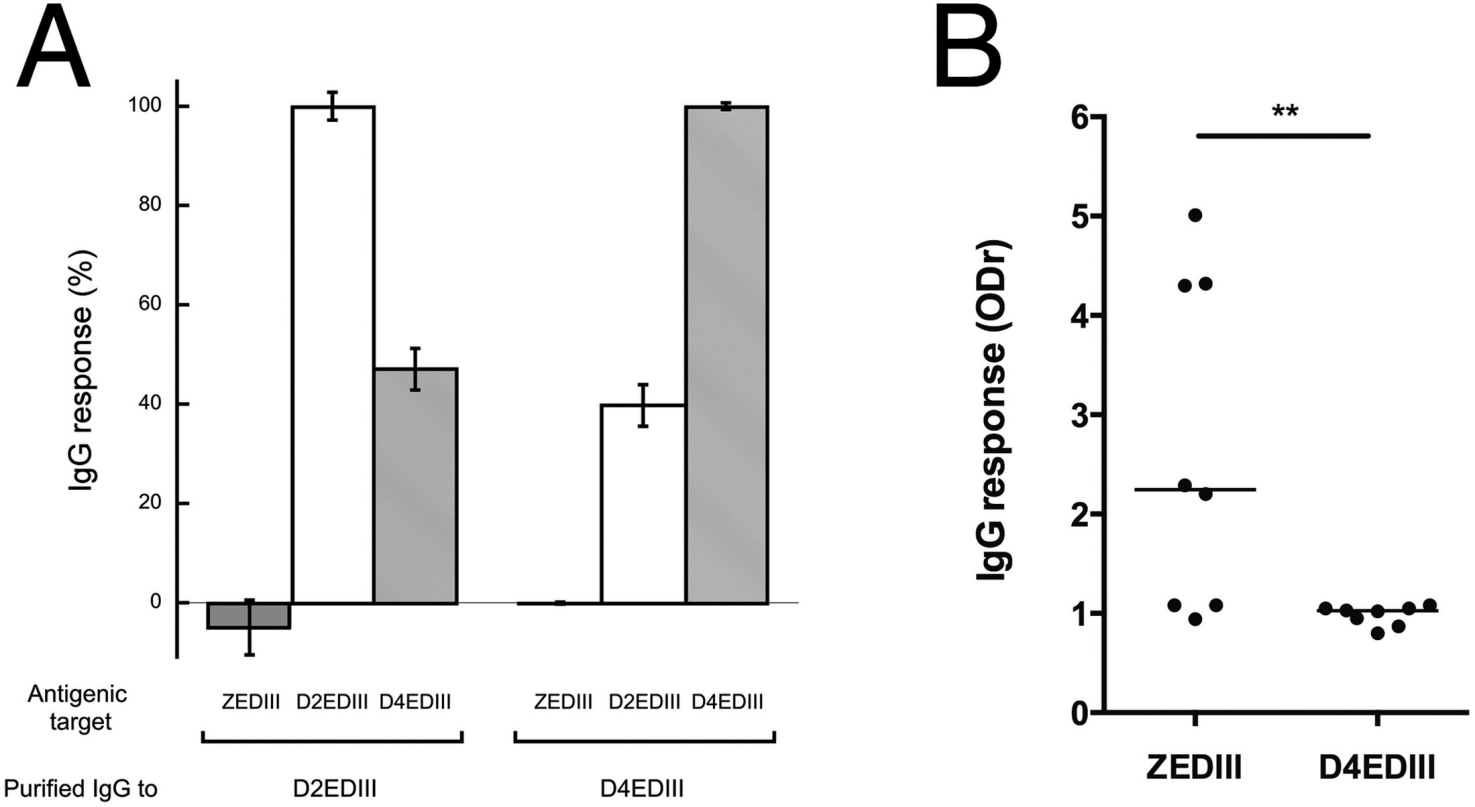
Cross-reactivity and specificity of IgG directed against ZEDIII. (A) Cross-reactivity of purified anti-EDIII IgG. Purified IgG against D2EDIII or D4EDIII were tested by ELISA. The recognition of ZEDIII is shown in dark grey, D2EDIII in white, and D4EDIII in light grey. Self-recognition is normalized to 100%. (B) The IgG response to ZEDIII and D4EDIII of immunized mice was measured by ELISA. The median is shown for both ZEDIII and D4EDIII. **W = 36, p = 0.0078.

We further characterized the lack of cross reactivity of anti-EDIII IgG of the three sub-groups selected from the ZIKV-positive and *flavivirus*-positive panels (Table 6). IgG purified against D2EDIII and D4EDIII cross-reacted with D4EDIII and D2EDIII (39.7 ± 4.2% and 47.0 ± 4.2% respectively, Fig 5A). Conversely, D2EDIII and D4EDIII-purified IgG did not recognize ZEDIII. ZEDIII-purified IgG recognized ZEDIII well (ODr = 15.9).

**Table 6:** Description of the sera origin used for the specific EDIII-IgG purification.

Groups purified on:	ZEDIII (ZIKV pos.)	D2EDIII (Flavivirus pos.)	D4EDIII (Flavivirus pos.)
Number, n	20	20	20
Gender, n women (%), n men (%), n ND (%)	16 (80%), 4 (20%)	12 (60%), 8 (40%)	15 (75%), 5 (25%)
Age in years, median, IQR, (range), n ND (%)	33, 30–42, (21–70)	50, 31–54, (6–75)	52, 33–67, (17–73), 1 (5%)
DPSO, median, IQR, (range), n ND (%)	29, 19–66, (12–139), 2 (10%)	45, 21–62, (8–90), 8 (40%)	32, 9–75, (8–99), 9 (45%)
n ZIKV sero-neutralizer (%), n ND (%)	12 (60%), 8 (40%)	-	-
n Positive recognition of ZEDIII (%), (ODr range)	20 (100%), (3.97–23.71)	0 (0%), (0.30–1.49)	0 (0%), (0.41–1.48)
n Positive recognition of D2EDIII (%), (ODr range)	8 (40%), (0.72–6.27)	20 (100%), (2.16–8.57)	9 (45%), (0.60–13.11)
n Positive recognition of D4EDIII (%), (ODr range)	14 (70%), (0.81–8.92)	10 (50%), (1.05–5.77)	20 (100%), (3.10–23.56)
Sera origins			
West Indies, n (%)	1 (5%)	-	-
Guadeloupe, n (%)	2 (10%)	9 (45%)	9 (45%)
Martinique, n (%)	13 (65%)	5 (25%)	11 (55%)
New Caledonia, n (%)	1 (5%)	-	-
Saint-Martin, n (%)	2 (10%)	6 (30%)	-
Venezuela, n (%)	1 (5%)	-	-

Abbreviations: ZEDIII, Zika virus envelope protein domain III; D2EDIII, Dengue virus serotype 2 envelope protein domain III; D4EDIII, Dengue virus serotype 4 envelope protein domain III; ND, not documented; DPSO, day post-symptoms onset; IQR, inter-quartile range; ODr, optical density ratio.

We next addressed the immune response induced by the recombinant ZEDIII. The median ODr of the sera obtained after the immunization of eight mice by ZEDIII was 2.25 and 1.02 against ZEDIII and D4EDIII, respectively. The difference between the ability of the sera to recognize the two recombinant proteins was significant (W = 36, p = 0.0078). None of the five ZEDIII-positive sera from mice (n = 8) recognized D4EDIII (Fig 5B), with the sequence closest to that of ZEDIII (Fig 2).

## Discussion

To date, no broad antibody cross-reactivity study has been performed for *flavivirus* with a particular emphasis on ZIKV, despite the often high co-endemicity of flaviviruses. Our study included more than 5,000 samples, providing sufficient power to observe statistically significant cross-reactivity results and select sera for the design of reference groups of patients. First, we observed low IgM cross-reactivity and high IgG cross reactivity against inactivated ZIKV, DENV, and WNV. This is generally observed with *flavivirus*-positive human sera, resulting in low diagnostic reliability of IgG-based assays [14, 24]. The use of peptides or recombinant proteins can partially improve the specificity of IgG-based diagnostic assays. Indeed, ZEDIII is specifically recognized with high sensitivity by anti-ZIKV IgG and can distinguish between DENV and ZIKV in late phase or post-infection. IgM detection shows relatively good specificity (only 23.8% cross-reactivity) permitting the determination of the infecting *flavivirus*, as already shown [25]. However, the IgM response does not persist. Thus, we studied ZEDIII recognition by IgGs, the most persistent antibodies.

We divided the set of sera into four groups based on various characteristics that allowed us to confirm their membership in each group. First, we determined the sensitivity of our assay using the reference group, the ZIKV-RT-PCR-positive group, which recognized the ZEDIII domain with the same sensitivity (90.7% [82.0; 99.4]) as the selected ZIKV-IgM-positive group (92.0% [84.5; 99.5]), allowing us to validate the ZIKV-IgM-positive group as a ZIKV-positive group. Thus, IgM that recognized only the total inactivated ZIKV, but not DENV (56.3% of ZIKV-infected patients), were induced by ZIKV, allowing the diagnosis of a recent ZIKV infection. Second, IgG from the sera of “*flavivirus”* patients, selected from 2013 to 2014 in Guadeloupe and Martinique, recognized all *flaviviruses* where and when ZIKV was likely not circulating. The large size of our group and the rational to select the sera allowed us to assemble relevant panels of samples suitable to support a robust and accurate study of ZIKV IgG detection using recombinant ZEDIII antigen.

The specificity for ZEDIII reached 89.9% and sensitivity 92.0%, whereas IgG presented cross-reactivity against total inactivated viruses (71% of IgG recognized both DENV and ZIKV). This makes recombinant EDIII one of the most robust tools for diagnosis [14, 24]. Of note, most of the 10 false-positive sera from the *flavivirus* groups gave a value close to the positive threshold. The ODr of only one serum sample was 6.26. Based on denaturation experiments (Fig 3), correct conformational folding of the EDIII protein is required for specific recognition. Indeed, chemical and heat denaturation of the protein led to the loss of recognition in ELISA assays, likely due to the loss of conformational epitopes, thereby decreasing specificity.

Given the high DENV seroprevalence observed in the area from which the ZIKV-IgM-positive group (mean age = 40 years) originated (93.5% of the Caribbean island population (mean age = 38 years), [26]) it is likely that they were infected with DENV prior to ZIKV infection, although we have no way of knowing whether the antibodies that recognized ZEDIII were actually induced by DENV. We thus purified IgG against immobilized EDIII domains to discriminate between IgG induced by and directed against ZIKV from that induced by DENV but cross-reacting to both ZIKV and DENV. No IgG from the selected pools purified against D2EDIII or D4EDIII recognized ZEDIII. Such a lack of cross-reactivity underscores the high specificity of the immune response against this antigenic domain. It is likely that the anti-ZEDIII antibodies were induced by ZIKV infection and not DENV infection, as we were unable to observe recognition of ZEDIII by the sera of patients who had never been infected by ZIKV. Thus, the high sensitivity obtained with our assay is due to ZIKV-induced IgG recognition and not potential cross-reactive IgG. The binding experiments developed to validate the low cross-reactivity in the ZEDIII-based ELISA showed that IgG purified against D4EDIII partially recognized D2EDIII, and vice-versa. This result is not surprising, as the experimental conditions were designed to force cross-reactivity further than in a classical sero-diagnostic test with high IgG concentrations. Instead, the experiment validates ZEDIII as a relevant antigen for sero-diagnosis. We further validated the high specificity of the humoral immune response against EDIII by assessing the ability of ZEDIII-immunized mouse sera to recognize recombinant EDIII. The sera of ZEDIII-positive mice did not recognize D4EDIII. As the D4EDIII amino-acid sequence is that closest to ZEDIII, it is possible that the result would be the same with any other *flavivirus* EDIII. Finally, we designed experiments using DEDIII-purified IgG to show that the observed sensitivity and specificity were due to only ZIKV-IgG binding to ZEDIII. The epitopes present on the surface of DEDIII were only recognized by IgG that did not recognize ZEDIII. IgG induced by ZEDIII immunization recognized only epitopes present on the surface of ZEDIII. Thus, the epitopes recognized by ZEDIII-IgG are highly different from those carried by D4EDIIIand the EDIII epitopes are virus specific. We hypothesized that WNV induced IgG will not recognize ZEDIII.

To date, the most robust and specific ZIKV sero-diagnostic test is the PRNT, which is costly and time-consuming. ELISA is also commonly used but usually serves as a first-line screen prior to PRNT, due to its low specificity. Indeed, we showed that 71% of IgG diagnoses were not reliable with an ELISA performed with inactivated ZIKV and that 48% of *flavivirus* sera were ZIKV-cross-reactive. We assessed our ZEDIII-based-ELISA and the NS1-based-ELISA (Euroimmun Assay) on the selected sera of the four groups. The ZEDIII- and NS1-based-ELISAs for IgG were 89.9% to 92.6% and 83.8% to 89.3% specific (calculated with the *flavivirus* group (n = 99) or the *flavivirus* group plus the negative group (n = 149), respectively), and had a sensitivity of 92.0% and 96.0%, respectively (Table 4). The sensitivity determined for the NS1-based ELISA varies significantly, depending on the cohort used: from 70.7% with the cohort of Matheus *et al*. [21] to 88.4% (ZIKV-RT-PCR-positive group) or 92.0% (ZIKV-IgM-positive group) (Z-test between the ZIKV-RT-PCR-positive or ZIKV-IgM-positive groups and the cohort of Matheus *et al*., p = 2.5257 and p = 4.4080, respectively). With our assay, the sensitivity varied non-significantly from 90.7% (ZIKV-RT-PCR-positive group) to 92.0% (ZIKV-IgM-positive group). The sensitivity of the ZEDIII-based ELISA can thus be considered stable. The ZIKV-IgM-positive group sera were collected in a DENV-endemic area, where the ZIKV infection is a second *flavivirus* infection, whereas this infection was the first for 65.6% of the ZIKV-RT-PCR-positive group. Thus, the difference in sensitivity could be due to this immunological scar.

Specificity is an important criterion for *flavivirus* diagnosis, as flaviviruses can induce cross-reactive IgG and high DENV prevalence is observed in the ZIKV endemic area. The specificity of the ZEDIII-based-ELISA was in the same range as that of the commercial test: within a 33.5% prevalence population, the probability of misdiagnosis would be 9.4% for the ZEDIII-based ELISA *versus* 12.1% for the NS1-based assay, which was not significantly different when considering the IC 95% overlap. However, unlike the NS1-based ELISA, the sensitivity of our assay did not depend on the cohort tested. Relative to the NS1-based ELISA, our assay would lead to the misdiagnosis of 1.9% additional positive patients but 7.1% fewer negative patients. The recombinant refolded ZEDIII domain could thus be a good target for diagnosis. According to Matheus *et al*. [21] and our study (data not shown), IgG raised against ZIKV and ZEDIII were still present, with at least 50% of the maximum ODr, after 300 DPSO and up to several years after infection [27]. This minor limitation could allow us to carry out retrospective seroprevalence studies of the ZIKV outbreak, whereas detecting ZIKV-induced IgG with ZEDIII could be used to detect recent past infections in populations at risk. A specific diagnosis must also be reliable for pregnant women or those planning to become pregnant to indicate their ZIKV immunization status, and not necessarily at the first stages of putative ZIKV infection, to orient medical care, as ZIKV can cause miscarriage [28, 29].

Recombinant EDIII proteins have already been used as IgG-targets in ELISA and Microsphere ImmunoAssay to detect the immune response of horses and humans in seroprevalence studies [30–33] and, in particular, ZEDIII to link pathology to infection in humans [34, 35]. However, no study has yet clearly shown the limits, specificity, and sensitivity of a ZEDIII-based ELISA. Our study reinforces precedent EDIII findings and shows the use of recombinant *flavivirus* protein-based ELISAs, such as that based on ZEDIII, to be potentially reliable, offering opportunities to develop rapid, inexpensive, and specific first-line assays. Antibodies specific to the infecting *flavivirus* are able to protect against a new infection by neutralization [13, 36], whereas cross-reacting antibodies have been extensively linked to antibody-dependent enhancement, increasing viremia [37]. This phenomenon has been observed for both DENV- and ZIKV-induced antibodies [38]. Our results suggest that ZEDIII, by inducing a strong and specific immune response, could also be a safe model for the development of vaccines.

## Supporting information

SD1 Fig STARD flow diagram for the selection of the three reference panels (ZIKV-IgM-positive, *flavivirus*, and negative). Abbreviations: NRC, National Reference Center; DENV, Dengue Virus; ZIKV, Zika virus; NS1, Non-Structural protein 1; ZEDIII, Zika virus envelope protein domain III; PRNT, Plaque Reduction Neutralization Titration; WNV, West-Nile virus; ESLV, Encephalitis Saint-Louis virus; TOSV, Toscana virus; RVFV, Ross Valley Fever virus; CHKV, Chikungunya virus. The STARD flow diagram for selection of the ZIFAG cohort has been described in the study of De Laval *et al*. [20].
(TIF)
