# Supplementary figures and images for: High specificity and sensitivity of Zika EDIII-based ELISA diagnosis highlighted by a large human reference panel
Source: PLoS Negl Trop Dis. 2019 Sep 20;13(9):e0007747. doi: 10.1371/journal.pntd.0007747 (PMC6774568; doi:10.1371/journal.pntd.0007747)

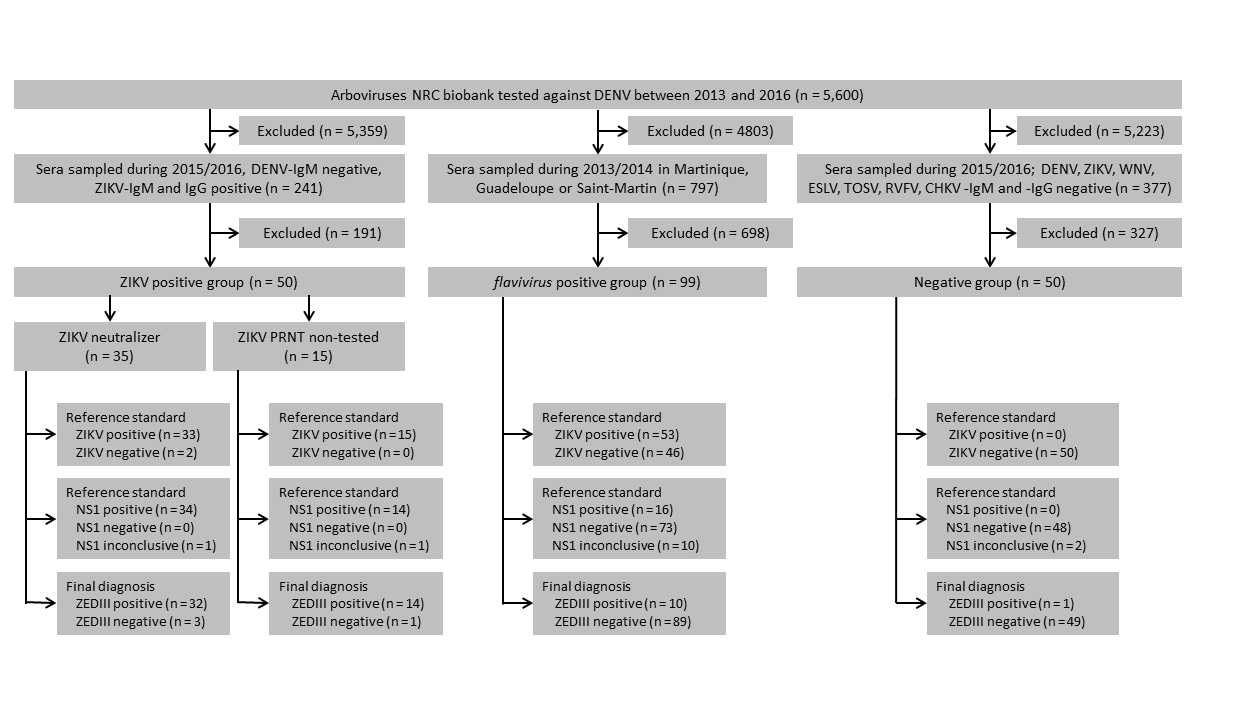

Supplement: SD1 Fig — Abbreviations: NRC, National Reference Center; DENV, Dengue Virus; ZIKV, Zika virus; NS1, Non-Structural protein 1; ZEDIII, Zika virus envelope protein domain III; PRNT, Plaque Reduction Neutralization Titration; WNV, West-Nile virus; ESLV, Encephalitis Saint-Louis virus; TOSV, Toscana virus; RVFV, Ross Valley Fever virus; CHKV, Chikungunya virus. The STARD flow diagram for selection of the ZIFAG cohort has been described in the study of De Laval et al. [20]. (TIF) [file pntd.0007747.s001.tif]
